# Relationships of physical activity and sedentary time in obese parent-child dyads: a cross-sectional study

**DOI:** 10.1186/s12889-016-2795-5

**Published:** 2016-02-06

**Authors:** Robert G. McMurray, Diane C. Berry, Todd A. Schwartz, Emily G. Hall, Madeline N. Neal, Siying Li, Diana Lam

**Affiliations:** 1 Department of Exercise & Sport Science, The University of North Carolina at Chapel Hill, Chapel Hill, North Carolina USA; 2 School of Nursing, The University of North Carolina at Chapel Hill, Campus Box 7460, Chapel Hill, North Carolina USA; 3 Department of Biostatistics, The University of North Carolina at Chapel Hill, Chapel Hill, North Carolina USA

**Keywords:** Accelerometry, Weekdays, Weekend days, Parents, Children obesity

## Abstract

**Background:**

Research suggests physical activity is linked to obesity. Further, the physical activity of healthy parents and their children is associated with each other. However, this relationship has not been examined in obese parents and their obese children.

**Methods:**

The purpose of this study was to compare the physical activity and sedentary time of obese, low-income, ethnic minority parents and their children on weekdays and weekend days using accelerometry. Data were obtained from eight rural sites in the middle and eastern part of North Carolina (N.C.), United States (U.S.) from 2007-2010 using a rolling enrollment. One hundred and ninety-nine obese parents (94 % female) and their obese children (54 % female) wore accelerometers simultaneously for three weekdays and one weekend day. Total physical activity, moderate-to-vigorous physical activity (MVPA) and sedentary time and proportions were determined.

**Results:**

Parents’ and children’s total physical activity and MVPA levels were lower on weekend days than weekdays. Total counts per minute for children on weekdays and weekend days were greater than for parents (p < 0.001). Total counts per minute were more highly correlated on weekend days than weekdays (r = 0.352, p < 0.0002 versus r = 0.165, p < 0.025). Parents’ performed MVPA for 14 (SD = ±25) and 9 (SD = ±16) minutes/day on weekdays and weekend days, respectively; children performed MVPA for 37 (SD = ±25) and 31(SD = ±38) minutes/day for weekdays and weekend days, respectively. Correlations between parents and children for MVPA were higher on weekend days versus weekdays (r = 0.253 and 0.177, respectively; p < 0.015). Associations for sedentary time followed a similar trend, with r = 0.33 (p < 0.0002) for weekend days and r = 0.016 (p < 0.026) for weekdays. Associations between obese parent-child dyads on sedentary time were stronger for girls, while associations between dyads on MVPA were stronger for boys. However, formal interaction analyses were not significant (p > 0.13).

**Discussion:**

Since physical activity levels of obese parents and their obese child are somewhat related, especially on weekend days, combined parent-child obesity programs focused on reducing sedentary time could be beneficial, particularly for the child.

**Conclusion:**

In conclusion, this study of the physical activity levels of obese parents and their obese children found some relationships between the parents’ and children’s physical activity and sedentary behavior patterns, especially on weekend days.

**Trial registration:**

NCT01378806.

## Background

The prevalence of overweight and obesity in adults and children has increased dramatically over the past three decades. Global obesity continues to rise; 39 % of adults over the age of 18 years were overweight and 13 % were obese in 2014 [1]. In addition, 42 million children under the age of 5 years were overweight or obese in 2013 [1]. Statistics from the U.S. published in 2014 indicates that 33.3 % of adults are overweight and 35.9 % are obese [2]. In addition, 18 % of U.S. children and adolescents (6-19 years) are overweight and 17 % are obese [2]. Further, obese parents are more likely to have obese children [3] and children who are overweight are more likely to be overweight or obese when they reach adulthood [2].

The link between obesity and physical inactivity or exercise is well established for both adults and children. In contrast, the link between parents and their children’s physical activity is not as well established; especially when both the parent and child are obese. For preadolescent children, parents have a dominant role in shaping the health behaviors of their children by serving as role models, creating a healthy home, and teaching and positively reinforcing their children’s efforts [4]. A number of studies have shown that parental modeling can contribute to the physical activity levels of children, particularly younger children [5–10]. However, support and encouragement for physical activity may be just as important [6, 11–20]. A previous study of healthy pre and early adolescent children [21] found no significant relationship between parents’ and children’s MVPA when both were in proximity of each other. Jago et al. [22] also found no significant relationship between parents and children’s MVPA levels. However, there was a weak, but significant association between parents and children for sedentary time (r ≤ 0.178, - 0.190), regardless of the gender of the child. Jago and associates [22] also pointed out that the weak relationship could be a function of the age of the children, because 10-11 year old children are establishing some degree of independence. Cameron and colleagues [23] using accelerometry in children and surveys of mothers found that some mothers modeled sedentary behavior for their children; however, they were unable to identify any modeling relationships for higher levels of physical activity [23]. Another study of mother and daughter obesity behaviors [24] found significant correlations between the dyads with regards to sedentary behaviors; but, they did not examine physical activity behaviors. Trost and colleagues [25], who used accelerometry to estimate physical activity of overweight children and a survey to obtain parental physical activity, reported no significant parental influences on children’s physical activity behaviors [25].

To date, little is known about the interactions between the physical activity behaviors of obese parents and their obese children. Furthermore, studies of obese parents and their obese children have not used simultaneous accelerometry measurement. This is important because questionnaires and surveys are somewhat inaccurate and only provide information on habitual activity, whereas accelerometry provides an objective view of present, and can even provide simultaneous activity levels. In addition, studies have also not examined the relationships between parent and child activity when divided into weekdays and weekend days, although Dunton partition their results into non-school hours [21]. The weekday and weekend day division may be an important factor, given the literature indicating differing activity levels between weekdays and weekend days for both adults [26, 27] and children [28–31]. Therefore, this study compared the levels of physical activity of obese children and one of their parents on weekdays and weekend days using accelerometry. We hypothesized that there would be significant relationships between children and their parents in amounts of sedentary time and amount of MVPA.

## Methods

The study design was cross-sectional and included baseline data (pre-randomization) from the Family Partners for Health Study [32, 33], which was a 5-year cluster randomized controlled trial for child and parent weight management. The experimental and control groups were pooled together for the analytic sample.

### Sample and Setting

A total of 44 or 45 overweight and obese parents or guardians and their overweight and obese children were enrolled in each of eight enrollment periods over 3 ½ years (2007-2010), for a total of 358 obese children and their 358 obese parents in rural middle and eastern N.C., U.S. [32]. Inclusion criteria for parents or guardians were ability to speak, write, and read English, a body mass index (BMI) >25 kg/m^2^, residing with a 2nd, 3rd or 4th grade child with a BMI > 85th percentile for age and gender, and consent to join the study. Inclusion criteria for children were ability to speak, write, and read in English, in the 2nd, 3rd, or 4th grade, 7 to 10 years of age, a BMI > 85th percentile for age and gender, residing with at least one parent or guardian with a BMI > 25 kg/m^2^, and assent and their parent or guardian’s consent to their participation. We chose 7-10 year old children because this age child is still quite dependent upon parental influences [22]. Parents and children were excluded from the Family Partners for Health Study if either had a heart murmur, congenital heart disease, a family history of sudden death, claustrophobia, or if they were participating in another weight management program [33].

In the Family Partners for Health Study [33], each site was randomized to either the experimental or control group the first time a group was enrolled, and the other condition was applied to the second enrolled group at that site. A total of eight elementary schools were used for recruitment and delivery of the intervention and data collection.

### Ethical Considerations

The study was approved by the University of North Carolina at Chapel Hill, N.C., U.S. , Institutional Review Board. All adult participants gave consent for themselves and their children and all children participants gave assent before enrolling in the study. Children were read the consent with their parent present. The consent was developed at a second grade level literacy level through the Institutional Review Board. All questions were answered before parents consented and children were asked to assent.

### Data Collection

All data were obtained at school sites where the programs were administered. Age of both the parent and child were computed from birthdate. Height and body mass were directly measured twice and averaged. Height in centimeters was measured using a stadiometer (Seca, Hanover, MD) and body mass in kilograms was measured using an electronic scale (Tanita WB-110A, Tanita, Arlington Heights, IL). BMI (kg/m^2^) was calculated by computer for all adult participants and BMI percentile was calculated for all child participants [34]. Physical activity levels were measured in both children and parents using the Actical accelerometer (Philips Respironics, Bend, Oregon, USA). The accelerometer was chosen because it is omni-directional and it is reliable in both adults and children [35–37], small, and waterproof; thus, it better captures activities that involve movements in many directions. Parents and children were instructed to wear the accelerometer on their right hip from Wednesday morning from the time they woke up until midnight on Saturday of that same week for a total of four days, which included three weekdays and one weekend day. Research assistants texted the parents each morning for the four days to remind them to put their and their children’s accelerometers on. The parents and children were instructed only to remove the accelerometer for sleep or bathing. They were provided with a prepaid envelope to return the accelerometers. Epochs for parents were defined as 60 seconds [38] and children were defined as 30 seconds. The shorter epochs for children were based on literature suggesting that their activities occur in shorter bouts than for adults [22, 39].

### Data Analysis

The Actical accelerometer data were first examined for compliance. Each obese parent-child dyad had to have simultaneous days of accelerometry wear to be included in the analyses. Data from 12:00 am hours to 05:00 am hours were removed, as were all data from night-shift workers. A day of data consisted of at least 10 hours on weekdays or 8 hours on weekend days; periods with no activity for longer than 60 minutes were considered as non-compliance [38]. For adults, minutes of light, moderate and vigorous physical activity were determined using the cut-points of 3 and 6 metabolic equivalents (MET) and algorithms developed in our pilot study [40]. For children, the moderate and vigorous cut-points used were developed by Colley et al. [41] while the sedentary threshold was developed by Puyau et al. [37]. Accelerometer thresholds are shown in Table 1.

**Table 1:** Accelerometry thresholds (counts/minute) for sedentary, light, moderate and vigorous activity levels for parents and children

Activity Level	Parents	Children
Sedentary	0 - 100	0 - 100
Light (<3 METs)	100 - 1535	100 - 1600
Moderate (3-6 METs)	1535 - 3960	1600 - 4700
Vigorous (>6 METs)	>3960	>4700

For accelerometry, means and standard deviations were computed for parents’ and children’s wear time, counts per minute and counts per hour, and proportions of the day spent being sedentary and participating in MVPA. The calculations were made separately for weekdays and weekend days. Accelerometry variables were then compared between parents and their children using paired t-tests. To determine the strength of the relationships between parents and their children on accelerometry variables, separate Spearman correlation coefficients were computed. In follow-up analyses, Spearman correlation coefficients evaluating the parent-child relationships were computed, stratifying by child’s age (continuously as well as stratified into 7-8 year olds versus 9-10 year olds, parent’s and child’s ethnicity (African American versus non-African American), and child’s gender. Additionally, separate general linear models were constructed for each of these factors (child’s age, parent’s and child’s ethnicity, and child’s gender), as well as parent BMI (treated continuously), to examine the interactions between parent accelerometry variables with the factors of interest, along with main effects; in these models the respective child accelerometry variables were treated as the dependent variable. The statistical tests of interaction terms were used to determine whether the relationship between child and parent accelerometry variables significantly varied by level of the interacting factor. All relationships were examined separately for weekdays and weekend days. Statistical Analysis Software (SAS) version 9.3 (Cary, N.C., U.S.) was used for all analyses, and statistical significance was set at the two-sided 0.05 level.

## Results

A total of 358 obese parents and 358 obese children were enrolled in the study; complete baseline accelerometry data were available on 199 parent-child dyads: 187 dyads (52 %) for weekdays and 109 dyads (30 %) for weekend days. Several dyads were not included in the analyses because the parent was a night-shift worker (n = 22) and others were eliminated because of incomplete accelerometry data (n = 137). Preliminary analysis indicated that the dyads used in the analyses were similar in BMI, gender, ethnicity, and child age to non-participants (p > 0.10). The mean age for parents was 37.5 ± 7.9 years, the majority were female (94 %), 50 % were married, 59 % were African American, and had a mean BMI of 37 ± 8 kg/m^2^. The children had a mean age of 9.0 (±0.9) years, were mostly female (54 %), African American (61 %), and in 3rd grade (42 %), and had a BMI percentile of 96 ± 5. Preliminary analyses found that there were no significant differences between the characteristics of the parents and children participating on weekdays versus weekend days (p > 0.05)

**Fig. 1:**
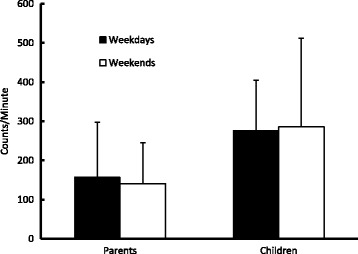
Mean (±SD) accelerometry counts per minute for parents and children on weekdays and weekend days

On weekdays the wear-time was an average (±SD) of 14.1 ± 1.6 hours per day for parents and 14.1 ± 1.4 hours per day for the children. The average counts per hour for parents on the weekdays was 9,449 ± 8,389 and the counts per minute was 157 ± 140, which was significantly less (p < 0.05) than for the children, who averaged 16,571 ± 7,766 counts per hour and a count of 276 ± 129 per minute on weekdays. On the weekend days, wear-time averaged 11.9 ± 2.6 hours per day for parents and 12.3 ± 2.6 hours per day for the children. The average hourly count for parents was 8,463 ± 6,239 and 141 ± 104 per minute; these averages were less than the weekday counts. The hourly counts for children per hour (17,160 ± 13,547) and per minute (286 ± 226) were slightly higher than weekday counts. For visualization only, the accelerometry counts per minute for parents and children are presented for weekdays and weekend days in Fig. 1.

The mean (±SD) percentage of time that parents were sedentary was 73 ± 11 % during weekdays, which was somewhat greater than the proportion of time for children (65 ± 10 %). On weekend days, the proportion of sedentary time did not appreciably change from weekday time (parents = 73 ± 12 % and children = 63 ± 14 %).

MVPA time was performed during only a small portion of the day for both obese parents and their obese children, with parents spending less awake time in MVPA than their children (parents: 1.6 ± 2.9 % for weekday and 1.2 ± 2.2 % for weekend days; children: 4.3 ± 3.0 % for weekday and 4.2 ± 5.2 % for weekend days). For these obese parents, these proportions represented a mean time of approximately 14 ± 25 minutes per day on weekdays and 9 ± 16 minutes per day on weekend days. For the obese children, average MVPA was performed for 37 ± 25 minutes per day on weekdays and 31 ± 38 minutes per day on weekend days.

Spearman correlation coefficients (r) between the parent-child dyads were weak (Table 2, column 2). Correlations between counts per minute for parents and children were stronger on the weekend days (r = 0.352, p = 0.0002) than on weekdays (r = 0.165, p = 0.025). The relationships between the proportion of the day spent in sedentary activities followed a similar trend, with r = 0.348 (p = 0.0002) for weekend days and r = 0.163 (p = 0.026) for weekdays. The correlations for MVPA level also followed a similar trend, being higher on the weekend days (r = 0.257; p = 0.007) than on weekdays (r = 0.192; p = 0.009).

**Table 2:** Exploratory Spearman correlations (rho) between the obese parent-child dyads presented overall and by age group, gender and ethnicity of the child

	Overall	Age group		Gender		Ethnicity	
		7-8 y	9-10 y	Girls	Boys	Non-AA	AA
Weekdays							
Cts/min	0.165^†^	0.135	0.179	0.148	0.197	0.252^†^	0.091
Sedentary (min)	0.163^†^	0.116	0.188	0.217^†^	0.083	0.271^†^	0.084
MVPA (min)	0.192^†^	0.197	0.184	0.164	0.255^†^	0.225	0.153
Weekend Days							
Cts/min	0.352^*^	0.205	0.474^*^	0.340^†^	0.351^†^	0.302^†^	0.351^†^
Sedentary (min)	0.348^*^	0.195	0.463^*^	0.326^†^	0.284	0.418^†^	0.203
MVPA (min)	0.257^†^	0.269	0.243	0.157	0.344^†^	0.256	0.235

*AA* African American, *y* years of age

*Cts/min* accelerometry counts per minute, *MVPA* moderate-to-vigorous physical activity

^†^p < 0.05

^*^p < 0.001

Some children’s characteristics affected these relationships (Table 2). For example, the correlation between parents’ and children’s counts per minute on weekdays was significant (p < 0.05) for non-African American children but not significant for African American children. On weekend days, the correlations were significant for both genders (p < 0.05), the older age group children (p < 0.05) of both genders, and both African American and non-African American children (p < 0.05), but not the younger children. The parent-child correlations for sedentary time were significant for girls (p < 0.05) and non-African American children (p < 0.05), and on weekend days for the older age group (p < 0.001). The correlations between MVPA levels of the parent-child dyads and weekdays and weekend days were strongest for boys (p < 0.05); correlations were not significant for the other subgroups. No significant interactions (p > 0.10) were found between the child’s gender, ethnicity or the parent’s ethnicity and counts per minute, proportions of sedentary time or MVPA on weekdays. However, the child’s age may have influenced the nature of the relationship for counts per minute on weekdays (p < 0.10). On weekend days, there was a significant interaction between the child’s age and counts per minute (p = 0.018 for continuous age) and proportion of sedentary time (p < 0.05 for age group).

## Discussion

The findings of this study support our hypothesis that there are significant relationships between obese parents and their obese children in both sedentary behavior and MVPA, with a stronger association on weekend days than weekdays. The weaker relationship during weekdays makes sense when one considers that on weekdays, children spend the majority (~60 %) of their waking hours away from their parents, at school, traveling to and from school, or in after-school activities. In support, Thompson et al. [42] found that during the week families spend little or no time together being physically active. The exploratory analyses also revealed that parent-child dyad relationships for sedentary time were stronger for girls, while the parent-child dyad relationships for MVPA were stronger for boys. Other studies also supported these results [15, 21, 42].

These findings are novel in that the entire sample consisted of obese parents and their obese children, were mostly minority (African American) with low-income, and were living in rural areas. In addition, accelerometry was used for simultaneous measurement of physical activity for both the parent and the child, and the results were determined by weekday versus weekend days. Previous studies have shown significant relationships between parents’ and their children’s activities, including both sedentary and MVPA [8, 13, 43], but not all studies agree [11, 12, 25]. The controversy could be related to differing sample sizes, differing locations (southeastern versus mid-western U.S. sites, urban versus rural settings), differing parental participation (mother or father), differing obesity status, and failure to separate weekdays from weekend days; although the literature clearly shows differences in physical activity for these two differing portions of the week [26, 28].

The relationships between the sedentary behavior and MVPA levels of our obese parents and their obese children were, in general, weak, accounting for only 2-12 % of the total variance. The relationships were stronger than those of Dunton et al. [21] or Jago et al. [22]; however, Dunton et al. [21] compared only activities completed with both parent and child in proximity of each other, while Jago and associates [22] used a sample of children older than ours. Conversely, our results are weaker than other previous reports of normal weight children and their parents [8, 43, 44]. Our results may reflect the fact that our sample were all obese individuals living in rural areas. From the parent’s perspective, some of the obese mothers may have had low perceptions of their ability to exercise and felt more obligated toward family responsibilities [22], which may have reduced their physical activity. In addition, many of the parents in our study mentioned concerns for their children's safety during exercise, suggesting that for obese children, factors other than parent modeling of the behavior are important; including enjoyment of physical activity, self-efficacy for physical activity, parental support, and availability of home physical activity equipment [11–13, 45]. However, accelerometry does not make it possible to determine the influence of these factors.

We found that similarities between obese parents’ and obese children’s physical activity behaviors were not associated with any characteristic of the parents that we explored (age, BMI, gender, or ethnicity), but were associated with certain characteristics of the children. Sedentary behavior correlations were strongest for girls and for non-African American children, who also had the highest levels of sedentary behavior. The literature on sedentary behaviors support stronger correlations between girls and parents than boys and parents [21, 22]. In addition, older children (9-10 year) on weekend days had lower activity levels than younger children, a fact also supported in the literature [15, 21, 22]. This could be related to older children asserting their independence or parents providing less supervision [46]. MVPA levels of boys appeared to be most influential in driving parent-child correlations for physical activity. The stratified correlations are particularly interesting in view of the fact that the vast majority of parents in this study were women. One could speculate that mothers who have sons tend to be more active than mothers who have daughters. However, this would need to be verified in a larger sample of obese parents and their obese children.

The weekday and weekend day patterns of activity were noteworthy. Previous studies of adults [26] and children [28, 29, 31] all suggest that there is less activity during weekends than on weekdays and that sedentary behavior increases on weekends. However, Trost and colleagues have suggested that the physical activity levels of children (in grades 1-6) actually increase on weekends [47]. Our data on the proportion of time spent in MVPA show similar percentages of time for both types of days for both obese parents (weekdays = 1.6 % and weekend days = 1.2 %) and obese children (weekdays = 4.3 % and weekend days = 4.2 %). However, total wear time was less on weekend days, resulting in fewer actual minutes of MVPA on weekend days than on weekdays for parents (weekend days = 9 minutes and weekdays = 14 minutes) and for children (weekend days = 31 minutes and weekdays = 37 minutes) (Fig. 1). Thus, our data are consistent with the majority of previous findings [26, 28, 29, 31].

An intriguing trend was observed in mean counts per hour, or the equivalent counts per minute (Fig. 1). As expected, parents had fewer counts per hour on weekend days (8,463) than on weekdays (9,449) and fewer minutes of MVPA on weekend days. In contrast, the counts per hour for children were actually a little higher on weekend days (17,160) than on weekdays (16,571), though they had lower proportions of MVPA. This suggests that on weekend days the children were spending fewer minutes in MVPA, but the time appeared to be spent at a slightly higher intensity. The percentages of time spent in sedentary behaviors did not change appreciably between weekdays and weekend days for either children or parents, but actual minutes of sedentary time decreased on the weekend days, probably related to less wear time and thus, fewer hours of recorded data on weekend days. Alternatively, the declines in both MVPA and sedentary minutes for both groups could suggest that slightly more time was spent in low intensity physical activity (LPA) on the weekend days than on weekdays. One can estimate the proportion of time spent in LPA by subtracting sedentary and MVPA from total time. In doing so *post-hoc* we noticed a little change in the proportion of time spent in LPA for both parents (25.8 % weekend days versus 25.4 % weekdays) and children (32.8 % weekend days versus 30.7 % weekdays). These estimated small differences suggest that the results were actually related to wear-time differences rather than an increase in LPA.

Overall, 50-80 % of the day was spent in sedentary behaviors by both parents and children, similar to the proportion reported by other research on adults [39] and children [39, 42]. The stronger relationship seen on weekend days than on weekdays is reasonable considering the time that children spend away from their parents and the home environment, during weekdays. These results suggest that the influence of obese parents on sedentary behaviors and MVPA of their obese children is greater on weekend days; thus, programs should consider putting greater emphasis on increasing parents’ MVPA not only on weekdays but also on weekends.

The 1-2 % of time the parents spent in MVPA was expected [26, 44]. However, we had anticipated that the parents would be more active on weekend days versus weekdays but the reverse occurred (~14 minutes on weekdays and ~9 minutes on the weekend days). One possible explanation could be that the majority of the sample had low-income jobs that were somewhat physically demanding during the week, causing them to be more fatigued on the weekend; thus, they had higher activity levels on weekdays than on weekend days. The children participated in more MVPA than their parents, as expected; but they also had fewer minutes of MVPA on weekend days than on weekdays; thus, there was some parallelism between parents and their children [48]. Although the children were more active than parents, these obese children’s activity levels fell short of meeting the guidelines of 60 minutes of MVPA per day [49] and were considerably less than those reported by some studies [18, 22, 28, 44]. These obese children are at risk for greater weight gain because of their low levels of MVPA.

This study had several strengths. First, it is one of the few studies that used accelerometry to objectively measure activity in both obese parents and their obese children on the same days. Second, the sample consisted of only obese parents and their obese children. Third, the study was one of a very few that have examined low socioeconomic status families from rural areas. Finally, the sample was larger than in most previous studies. There were also a few limitations. The proportion of the total sample with complete accelerometry data for both parent and child was less than expected (56 %), though the dyads that had complete data were similar in gender, age and BMI status to the other participants. A concern may be that 94 % of the parents were female, which could have biased the generalizability of the findings to all parent-child dyads. Thus our findings may be better suited for mother-child relationships. Previous studies have reported a similar issue [15, 21, 22, 50]. Finally, other data were not obtained on other factors, like home environment, geographical environmental issues, and enjoyment of physical activity, which could have provided more insight into the reasons for the low relationships, particularly since gender, ethnicity, and BMI of the parent or child minimally influenced the relationships. Therefore, these other factors could be considered biases in this study focusing on baseline accelerometer data. Suggestions for future studies include the need to measure both parents and children for a full seven days and include an assessment of the home environment, geographical environmental issues, and parent and child enjoyment of physical activity. This could provide additional information that could assist in designing future studies.

## Conclusions

In conclusion, this study of the physical activity levels of obese parents and their obese children found some relationships between the parents’ and children’s physical activity and sedentary behavior patterns, especially on weekend days. These relationships were not generally associated with the age, gender, ethnicity, or BMI of the parent, suggesting that obese parents, regardless of physical characteristics, model physical activity levels for their obese children. However, the results were dependent upon the age, gender, and ethnicity of the child. Thus, if both the child and parent are obese, combined obesity treatment programs could be beneficial, particularly for the child. The results also suggest that the influence of parents on sedentary behaviors and MVPA levels of their children is greater on weekend days; thus, reducing the sedentary behaviors and increasing MVPA levels of parents on weekends could have some impact on the behaviors of their obese children.

## Data Availability

The data can be obtained by contacting the corresponding author Dr. Diane Berry by email.
